# Philadelphia Academy of Surgery

**Published:** 1881-05

**Authors:** 


### PHILADELPHIA ACADEMY OF SURGERY.

*Atrophy of Muscles and Bones Consecutive to Club-Foot.*—Dr. T. G. Morton presented two patients showing atrophy of the limbs from club-foot, in substantiation of his statement, made at a previous meeting, that the bones were diminished in acquired and congenial talipes, and that the affected leg never kept pace with the sound one.

D C., aged 7 years, at the age of twenty months was suddenly seized with infantile palsy which involved the left lower extremity; he was in bed rather more than a week, and after this dragged the limb. In time it improved, and gradually he became able to use it freely. The limb has, however, never grown in the same proportion as the sound one. The child, as a result of this attack of palsy of the anterior leg muscles, has an acquired equinus, due to the unbalanced power of the calf muscles. Measurement shows differences in the circumference of the limbs and in the length of the bones. The left lower extremity is an inch shorter than the right, and one and a quarter inches less in girth below the knee. Around the point of the heel and the top of the foot the difference in measurement is a half inch.

The second case was one of congenital club foot with the following history. Six weeks after birth he was operated on for equinovarus, and had subsequently a "sore foot," which prevented his wearing the apparatus prescribed. At six years of age a second operation was performed, which was also followed by a "sore foot." At the present time the bones of the left leg are smaller than those of the other; and careful measurement gives the following differences in the circumference of the limb at various points and in the length of the leg and foot:

The left is one inch less than right at top of thigh; two and one-half inches less at middle of thigh; three and three-quarter inches less below knee; one and one-quarter inch less around heel; one and three-quarter inch less in length of foot; and one inch less in its entire length.

*Upward Dislocation of the Acromial end of the Clavicle.*—Dr. S. W. Gross exhibited a gentleman suffering with this injury, caused by a fall, for whom he had had constructed an apparatus to prevent displacement of the joint-surfaces, which are retained in position with such difficulty after this dislocation. The elbow fitted into a sort of cup, from which a strap of webbing passed over the shoulder and by means of a pad made pressure upon the outer end of the clavicle. Other bands were required to prevent this primary one from slipping. The bone seemed to be in good position at the time of exhibition, which was five weeks after the accident. The apparatus was as efficient as strips of adhesive plaster, or the plaster-of-Paris bandage, and had advantages in regard to cleanliness and convenience.

Dr. Levis had thought of treating such cases, like fracture of the patella, by hooks or by wiring.

Dr. O. H. Allis stated that wiring had been done successfully by a surgeon in San Francisco

*Scrotal Tumor.*—Dr. R. J. Levis brought to the notice of the Academy a patient having a large, painless, evidently non-malignant tumor of the right side of the scrotum. It had existed seven years and was inconvenient only from its weight. On palpation it felt much like them ammary gland. Dr. Levis believed it to be a fibro fatty growth, not connected with the cord or testicle, and probably outside of the vaginal tunic.

Dr. S. W. Gross believed it to be a fibro-fatty tumor of the spermatic cord, which had grown downwards. As far as his knowledge went, it was not likely to be an intrascrotal growth without being connected with the cord. Myomata, sarcomata, and fibromata are the forms of tumor that occur in the scrotum.

*Traumatic Hernia of the Bladder.*—Dr. Morton presented a patient with traumatic hernia of the bladder, and read the following history of the case:

Jacob S., aged 34, single, was admitted into the Pennsylvania Hospital January 26, 1881. At the age of ten years he had sustained, by a machinery crush, a compound and comminuted fracture of the right pelvis, with dislocation and fracture of the head of the femur on the same side; the wound which communicated with the broken illeum extended from the anterior superior spinous process along the line of the groin and perineum, and involved the base of the bladder and outlet of the rectum; immense sloughing followed, from urinary infiltration, and subsequently erysipelas was developed. From the time of the accident up to 1868, fragments of the bone continued to be thrown off, and the major portion of the pubis came away entire, as did also the head of the femur, split, however, in three pieces; consolidation of the shaft of the bone took place at an angle, on the dorsum ilii, with great shortening of the limb and immense atrophy; the wound in the groin and perineum closed up, except near the rectum, where a portion of the bladder protruded soon after the injury and has so continued to the present time, and from which opening all the urine is discharged, none having since the injury passed through the urethra; the outlet of the rectum in front is somewhat constricted by a band of tissue which extends across the perineum; but behind this ridge of skin the bladder and rectum open into each other.

The patient has since his recovery enjoyed the best of health, and is engaged in laborious work. An examination of the urethra shows it to be patulous to the neck of the bladder where the urethra is abruptly cut off, and at this place in the perineum the rupture of the bladder occurred. The patient is able to retain most of the urine, and does not suffer at all from incontinence, from the fact that the bladder falls forward and to the right side and fills up the space formerly occupied by the bony pelvis. He has no difficulty with the bowels unless when suffering from diarrhœa. He has ordinary venereal desire, but the discharges of semen pass through the vesical opening in the perineum. No operation was deemed advisable.

*Death after Sounding for Vesical Calculus.*—Dr. William Hunt showed a bladder and calculus removed from a man aged 18 years, who had died at the Pennsylvania Hospital. The patient was admitted with symptoms of stone in the bladder, and was generally in a poor condition of health; he had never been sounded. The resident surgeon introduced a sound and discovered the calculus. On the day after the patient's admission, before Dr. Hunt had made his visit, death took place, without the occurrence of chill or fever, but apparently from profound nervous shock. The autopsy was partial, as permission could not be obtained to examine the kidneys or chest. The patient, however, presented no œdema of the legs, nor had he any convulsive seizures.

Dr. T. G. Morton thought it possible that the death had no connection with the examination of the bladder with the sound, but occurred from exhaustion due to prolonged suffering. He knew of an instance of death taking place after catheterization; but then chill and other symptoms of urethral fever had presented themselves.—*Medical Times.*

